# Supplementary material for: Harnessing the Role of Bacterial Plasma Membrane Modifications for the Development of Sustainable Membranotropic Phytotherapeutics
Source: Membranes (Basel). 2022 Sep 22;12(10):914. doi: 10.3390/membranes12100914 (PMC9612325; doi:10.3390/membranes12100914)
Supplement: Supplementary file 1 [file membranes-12-00914-s001.zip › membranes-1890375-supplementary.pdf]

Supplementary Information

Table S1. Modification of outer membrane lipids in Gram negative bacteria leading to drug resistance.

Bacteria	OM modification	Effect on bacterial phenotype	Effect on virulence/antibiotic resistance	References
		**Fatty acylation of lipid A**		
B. bronchiseptica	Removal of palmitoyl group at C-3'.	Adaptation to changing host environment.	Persistence in colonization of mouse respiratory tract	[142]
B.parapertussis	Addition of palmitate to the C2' and C3′.	Reducing membrane fluidity and maintaining OM integrity.	CAMPs (C18G)	[52]
E. coli	Addition of palmitate to lipid A.	Reduction in membrane permeability.	β-lactam antibiotics, gramicidin	[49]
A.baumanii (MAC204)	Palmitoylation of lipid A.	Stabilization of the LPS leaflet and decreased electrostatic interactions with cations.	CAMPs	[58]
Enterobacter	Removal or palmitoylation of *N*-linked *R*-3-hydroxymyristate chain at position 2 of lipid A.	Interfere with CAMP translocation across the bilayer.	CAMPs	[51]
S.typhimurium	Palmitoylation of PG and CL in OM.	Decreased permeabilityof OM.	C18G	[54,55,143]
B.subtillis	Hepta-acylated lipid A.	Increased packing and reduced outer membrane permeability.	C18G (10µg/ml)	[46]
Bordetella	Palmitoylation of lipid A at 3'.	Changes the fluidity of the OM.	CAMPs	[51]
K.pneumoniae	Secondary acylation of lipid A with myristate.	Altering the activation of the Toll-like receptor 4 (TLR4)/MD-2 receptor complex.	polymixin B and colistin	[48]
V. cholerae	Glycine modification at 3'-OH of lipid A**.**Addition of glycine or diglycine residue to a 3′- linked acyl chain of lipid A.	Decrease in membrane fluidity and negative surface charge.	Polymyxin B (85-100 fold increase in MIC)	[144,145]

B.pertussis	Modification of phosphates of lipid A with positively charged GlcN groups	Stabilization of OM.	LL-37	[146]
C.jejuni	Addition of three amide-linked acyl chains and only one ester-linked acyl chain instead of two ester- and two amide-linked acyl chains to lipid A.	Increased membrane stability and decreased permeability.	Polymixin B	[57]
S.typhimurium	Addition of amino sugars or phosphoethanolamine to phosphate group of lipid A.	Reduction in negative charge of the molecule by removal or substitution of the negatively charged phosphate groups at the 1- and 4′- positions.	Polymyxin B	[144,147]
C.jejuni	Modification of lipid A with phosphoethanolamine residue at 1′ and 4′ positions.	Masking of phosphate groups.	CAMP (20 fold increase in resistance)	[148]
S.enterica	Deacetylation of lipid A and addition of cationic PE to lipid A.	Decreased membrane fluidity and surface charge.	Colistin	[149]
F.tularensis	Deacetylation of lipid A.	Increasing surface charge (2-fold)	Polymixin B	[150]
A. baumanii	Ethylonamine, aminoarabinose and D-galactosamine modified lipid A.	Lipid A acylation, phosphorylation, and glycosylation.	Colistin (24µg/ml) and polymixin B	[151]
S.typhimurium	Addition of Ara4N to lipid A.	Increase in cationic charge.	Polymyxin (2fold increase in MIC)	[42]
A.baumanii	Complete abolishment of Lipopolysaccaride.	Reduced binding of antibiotics due to altered permeability.	Colistin having 128 (µg/ml) fold increase in MIC.	[36]
**Modification of core oligosaccharides**				
S. enterica	Phosphorylation of lipid A.	LPS core modification leading to lower permeability.	Polymixin B	[67]
A.. baumannii	Oligosaccharide head-group interactions with divalent cations.	Increased stability and rigidity of OM by liquid crystalline transition.	Polymyxin B	[56]
C. jejuni	Modification in a heptose sugar of the core oligosaccharide charge repulsion.	Decreased membrane fluidity or charge repulsion.	Polymixin B and Colistin	[152]

Abb.: GlcN,Glycine; PE, Phosphatidylethanolamine; L-Ara4N,4-amino-4-deoxy-L-arabinose.

Table S2. Phospholipid modification in bacterial membrane leading to drug resistance.

Microorganisms	Membrane component	Membrane properties	Antibiotic (µg/ml)	References
S.aureus	35% increase in Lys-PG.	25% increase in cationic charge.	Gallidermin (0.5 µg/ml) and Daptomycin (1.8 µg/ml)	[71]
S. aureus	Gain-of-function mutations in MPRF.	Increasing surface charge (24.5 ± 5.6)%	Daptomycin (>2µg/ml) 2 fold increase in MIC.	[85]
MRSA	1.2 fold increase in Lys-PG.	Modification in membrane surface charge dueto1.52- increase in total L-PG.	Polymixin B, (30µg/ml) Vancomycin	[70]
B. licheniformis	Increase in lys-PG.	Increase in cationic charge.	CAMPs	[73]
S. .typhimurium	Palmitoylatedacylphosphatidylglycerols.	Reduction in PG polarity and increased hydrophobicity and saturation of the bilayer.	CAMP	[54]
C.perfringens	Modification of PG with Alanine.	Reduce negative surface charge.	CAMP (defensin)	[76]
S.aureus	Co-expression of the alanyl-PG (Ala-PG) synthase with flippase domains of Lys-PG synthesizing MPRF proteins.	13% increase in overall aminoacyl phospholipid content.	Daptomycin(6-fold increase) gallidermin and nisin(4-fold)	[77]
B. subtilis	Reduction of the PG content as a consequence of PG modification with lysine.	Reducing negative charge.	Daptomycin resistance	[154]
S.aureus	Release of Phospholipid.	Inactivation of antibiotic.	Daptomycin resistance	[155]
P. aeruginosa	Phospholipid incorporation of exogenous fatty acids such as arachidonic acid docosahexaenoic acid.	Decreased membrane Permeability upto>10%.	Polymyxin B (8-fold increase in MIC) and Colistin (two-fold increase in MIC)	[156]
S. aureus DAPRSA	D-alanylation of teichoic acid.	15% increase in D-alanylation of teichoic acid.	Imipenem (16 µg/ml)	[157]
S. aureus	D-alanylation of teichoicacid.	93% increase in TA, 13% increase in cationic charge	Daptomycin (8µg/ml)(32 fold increase in MIC)	[158]
P. aeruginosa	Formation of Cardiolipinmicrodomains.	Increased membrane stability.	Aminoglycosides (diNn)] MIC-4µg/ml	[98]

E. coli	Increase in CL content in formation of microdomain	Prevent membrane binding.	Magainin 2, polybia-MP1, LL-37, and ΔM2	[95]
E. coli	Increase in CL helps in enhanced transport of lpxM adding acylation.	Overexpression of Cls A increases antibiotic resistance.	Vancomycin(256 µg/mL)1.3 fold increase in MIC	[60]
P. putida	Increase in CL content.	Enhances membrane rigidity.	Chloramphenicol (250 mg/ml)and tetracycline (1.8 mg/ml) (4 fold increase in MIC_{50})	[159]
E. coli	In presence of CL, AcrZ and AcrB export antibiotics.	Prevent antibiotic binding.	Chloramphenicol	[160]
P. aeruginosa	Cardiolipin relocation and clustering.	Changes in fusion, permeabilization and hydration of membrane.	3'6-dinonylneamine(3'6 diNn)	[99]
E. coli	Depletion or lack of CL content.	Increasingly liquid-disordered reducing membrane permeability.	Sphingosine	[100]
Liposome (PC:PG)	CL restricts daptomycinto the outer membrane leaflet.	Diminish bilayer permeability.	Daptomycin	[87]
S.aureus	Single amino acid substitutions in cardiolipin synthase 2 led to increased CL biosynthesis.	Prevent membrane disruption,reduced neutrophil chemoattraction to infection site.	Daptomycin	[161]
S.aureus	CL inhibits translocation of daptomycin to the IM leaflet.	Prevents membrane translocation and permeabilization.	Daptomycin	[87]
LUV (PG:PC ,DOPC)	Increase in CL content.	Weaken daptomycin binding.	Daptomycin	[162]
S.aureus	Increased CL syntheses reduce PG content.	Decrease in net negative charge of membrane.	Daptomycin	[163]
MRSA	Formation of Staphyloxanthinmicrodomains.	Increase membrane rigidity.	Oxacillin(6µg/ml) Flucoxacillin(4µg/ml), Naficillin (4µg/ml), Dicloxacillin(5µg/ml) Ampicillin(10µg/ml) and Methicillin,	[80,164]
MRSA	Staphyloxanthin and its derivatives condense as the constituent lipids of functional membrane microdomains (FMM).	Maintaining membrane integrity.	Penicillin	[92]

Abb.: MPRF, Multiple Peptide Resistance Factor; CL,Cardiolipin;DAPRSA, Daptomycin resistant *S.aureus*; TA, Teichoic acid; CAMPs, Cationic antimicrobial peptide;
